# Suppression of the PI3K Pathway In Vivo Reduces Cystitis-Induced Bladder Hypertrophy and Restores Bladder Capacity Examined by Magnetic Resonance Imaging

**DOI:** 10.1371/journal.pone.0114536

**Published:** 2014-12-08

**Authors:** Zhongwei Qiao, Chunmei Xia, Shanwei Shen, Frank D. Corwin, Miao Liu, Ruijuan Guan, John R. Grider, Li-Ya Qiao

**Affiliations:** 1 Children's Hospital of Fudan University, Division of Radiology, Shanghai, China; 2 Department of Physiology and Pathophysiology, Shanghai Medical College, Fudan University, Shanghai, China; 3 Department of Physiology and Biophysics, Virginia Commonwealth University School of Medicine, Richmond, Virginia, United States of America; 4 Department of Radiology, Virginia Commonwealth University School of Medicine, Richmond, Virginia, United States of America; 5 Department of Internal Medicine, Virginia Commonwealth University School of Medicine, Richmond, Virginia, United States of America; Oklahoma University Health Sciences Center, United States of America

## Abstract

This study utilized magnetic resonance imaging (MRI) to monitor the real-time status of the urinary bladder in normal and diseased states following cyclophosphamide (CYP)-induced cystitis, and also examined the role of the phosphoinositide 3-kinase (PI3K) pathway in the regulation of urinary bladder hypertrophy in vivo. Our results showed that under MRI visualization the urinary bladder wall was significantly thickened at 8 h and 48 h post CYP injection. The intravesical volume of the urinary bladder was also markedly reduced. Treatment of the cystitis animals with a specific PI3K inhibitor LY294002 reduced cystitis-induced bladder wall thickening and enlarged the intravesical volumes. To confirm the MRI results, we performed H&E stain postmortem and examined the levels of type I collagen by real-time PCR and western blot. Inhibition of the PI3K in vivo reduced the levels of type I collagen mRNA and protein in the urinary bladder ultimately attenuating cystitis-induced bladder hypertrophy. The bladder mass calculated according to MRI data was consistent to the bladder weight measured ex vivo under each drug treatment. MRI results also showed that the urinary bladder from animals with cystitis demonstrated high magnetic signal intensity indicating considerable inflammation of the urinary bladder when compared to normal animals. This was confirmed by examination of the pro-inflammatory factors showing that interleukin (IL)-1α, IL-6 and tumor necrosis factor (TNF)α levels in the urinary bladder were increased with cystitis. Our results suggest that MRI can be a useful technique in tracing bladder anatomy and examining bladder hypertrophy in vivo during disease development and the PI3K pathway has a critical role in regulating bladder hypertrophy during cystitis.

## Introduction

The urinary bladder is constituted by four basic layers of tissues, namely the urothelium, the suburothelium space, the detrusor smooth muscle layer, and the outermost serous membrane. The urothelium layer acts as a permeability barrier protecting underlying tissues against noxious urine components. The lamina propria is rich in nerves, blood vessels, connective tissues, and also contains a variety of immune cells. In response to noxious stimuli or injury of the urinary bladder, destruction of the urothelium architecture occurs which is accompanied by enhanced vasodilation, and accumulation and infiltration of immune substances thereby causing excessive release of inflammatory mediators, erythematous swelling and hemorrhage of the bladder [1], [2], [3], [4], [5]. Dysfunctional pathology of the smooth muscle layer in the bladder wall is tightly related to poor compliance of the urinary bladder and detrusor instability which is often attributable to bladder wall thickening caused by excessive deposition of fibrotic connective tissues and detrusor smooth muscle hyperplasia and/or hypertrophy [1], [6], [7]. In inflammatory state, the serous membrane may also become thickened with subserous cellular tissue infiltration.

The urinary bladder wall thickening is often seen in patients and animals with cystitis, bladder outlet obstruction (BOO), and sometimes with neurological disorders [6], [8], [9], [10]. Previous studies with an animal model of cystitis induced by intraperitoneal injection of cyclophosphamide (CYP) or intravesical instillation of acrolein, a metabolite of CYP [11], demonstrate that the weight of the urinary bladder is dramatically increased in the diseased animals when compared to healthy controls [6], [12], [13]. Several factors are suggested to have critical roles in bladder pathology during chemically induced cystitis. These factors include but are not limited to growth factors such as nerve growth factor (NGF) [14], [15] and transforming growth factor-beta (TGF)β [14], [16], cannabinoids [17], [18], [19], cytokines and chemokines [16], [20], [21], [22], muscarinic and purinergic systems [23], [24], [25], [26], and a variety of inflammatory mediators [27], [28]. The cellular responses of these factors are mediated by specific receptors such as receptor tyrosine kinase (RTK) or G-protein coupled receptor (GPCR), and can converge on the PI3K and Akt pathways [29], [30], [31], [32]. In turn, activation of the PI3K/Akt pathway also leads to gene expression and cellular growth and survival [33], [34]. Previous studies by us and others show that in CYP-induced cystitis the activity of Akt is increased in the urinary bladder [6], dorsal root ganglia [35], and spinal cord [36]. Inhibition of the PI3K-mediated Akt activation reverses cystitis-induced spinal central sensitization [36] as well as bladder overactivity examined by cystometry [37]. In the understanding of molecular mechanisms underlying the regulation of bladder hypertrophy, the role of the PI3K/Akt pathway has not been investigated and is the focus of this study.

Magnetic resonance imaging (MRI), also called magnetic resonance tomography, is widely used in clinical settings to investigate the anatomy and function of the body in both health and disease. MRI has been widely used for visualization of internal structures in the body. In comparison with computed tomography (CT) scan, MRI is better in determining the depth of wall invasion in bladder tumors [38] with the greatest advantage in differentiating between a normal bladder and other pathologic conditions including inflammatory and congestive processes [39]. In humans, MRI is used to diagnose bladder hypertrophy caused by BOO or cystitis [40], [41]. In a recent pre-clinical study T2-weighted MRI is used to visualize the rat bladder with partial BOO before and after mesenchymal stem cell transplant [42], which shows that bladder wall thickness is correlated with the expression level of collagen and TGF-β protein in the urinary bladder [42].

The present study was undertaken to examine the role of the PI3K inhibition in urinary bladder hypertrophy caused by cystitis by combining an in vivo approach of MR imaging and ex vivo approaches of histological and molecular analysis. Excessive cellular growth, fibrosis and inflammation are key contributing factors causing bladder hypertrophy in cystitis. The PI3K/Akt pathway functioning as a master switch in mediating multi-signaling networks may govern the growth promoting processes thereby regulating bladder hypertrophy.

## Materials and Methods

### Experimental animals and ethics statement

Adult male rats weighing 150–200 g were used for all studies. All experimental protocols involving animal use were approved by the Institutional Animal Care and Use Committee at the Virginia Commonwealth University (IACUC # AM10315), and at the Fudan University. Animal care was in accordance with the Association for Assessment and Accreditation of Laboratory Animal Care (AAALAC) and National Institutes of Health guidelines. All efforts were made to minimize the potential for animal pain, stress or distress as well as to reduce the number of animals used.

### Induction of cystitis

Cystitis was induced in rats by intraperitoneal injection of cyclophosphamide (CYP, Sigma-Aldrich, St. Louis, MO) at a single dose of 150 mg/kg body weight. The animals were allowed to survive for 8 or 48 hours (h). In MRI studies, each rat served as its own control before and after CYP treatment. For other studies, control rats received volume-matched injections of saline. All injections were performed under isoflurane (2%) anesthesia.

### Drug treatment of animals

To block the PI3K/Akt pathway in vivo, an intraperitoneal injection of a PI3K inhibitor LY294002 (Calbiochem, dissolved in DMSO as stock and diluted in saline for injection) at a single dose of 50 µg/kg body weight was made immediately after CYP injection [36]. The same amount and concentration of DMSO was used as vehicle control.

### Magnetic resonance imaging of the urinary bladder

All MRI measurements were acquired utilizing the Siemens Avanto 1.5 T scanner. The imaging coil was a phased array coil for rat with inner diameter of 5 cm. Under anesthesia (2% isoflurane), the animal was placed in the supine position into the MRI built-in chamber for scanning. A spin-echo T2 weighted plus water suppression MRI sequence was used with parameters: TR 7500 ms, TE 124 ms, TI 2200 ms, FOV 120 mm^2^, and slice thickness 2 mm. The images were obtained in the axial and sagittal planes according to procedures modified from the method used in human MRI to diagnose bladder hypertrophy caused by bladder outlet obstruction (BOO) or cystitis [40]. It was shown that it was not necessary to manually empty the urinary bladder (manually squeezing bladder often causes wall collapse) to evaluate bladder wall thickening [40]. After the images were obtained, the outer diameter of the bladder wall (2× tR: total radius), the inner diameter of the bladder wall (2× iR: intravesical radius), and the thickness of the wall (direct measurement or tR - iR) were measured with built-in software. Each parameter was measured at 4 directions (horizontal, vertical, two diagonal) and averaged. The bladder mass was calculated by subtracting the intravesical volume from the total vesicle volume and then multiplied by the urinary bladder density shown as 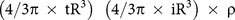
where tR was total radius, iR was inner radius, and ρ was the density of the urinary bladder which was considered as 1 [43].

### RNA extraction and quantitative real-time PCR

Total RNA was extracted using a RNA extraction kit RNAqueous (Ambion, TX). RNA concentration was determined spectrophotometrically. cDNA was synthesized using High Capacity cDNA Reverse Transcription Kit (Applied Biosystems, ABI). Following reverse transcription, quantitative real-time PCR was performed on StepOnePlus Real-Time PCR Systems (Applied Biosystems, ABI) under a condition of 40 cycles of 95°C for 15 s and 60°C for 1 min, using SYBR Green as indicator. The level of target mRNA was normalized against the expression of the internal control 18S in the same sample that was calculated with ΔCt method. The sequences of primers were listed in Table 1. The expression level of target mRNA in control group from each independent experiment was considered as 1, and the relative expression level of target mRNA in experimental groups was adjusted as a ratio to its control in each independent experiment and expressed as fold changes (2^−ΔΔCt^-fold).

**Table 1:** Primers used in real-time PCR.

TNFα F(5′-3′) AGCCCGTAGCCCACGTCGTA
TNFα R(5′-3′) ATGCCATTGGCCAGGAGGGC
IL-1α F(5′-3′) CCGCAGCTTTCCCAGAGCTGTT
IL-1α R(5′-3′) TCATGGAGGGCAGTCCCCGT
IL-6 F(5′-3′) TGTTGACAGCCACTGCCTTCCC
IL-6 R(5′-3′) ACTGGTCTGTTGTGGGTGGTATCCT

### Western blot

The urinary bladder was freshly dissected out and homogenized in T-per buffer (Pierce Biotechnology, Rockford, IL) supplemented with protease and phosphatase inhibitor cocktails (Sigma). The protein extracts were subject to centrifugation at 20,200 g for 10 min at 4°C, and the supernatant was removed to a fresh tube. The protein concentration was determined using Bio-Rad DC protein assay kit. Proteins were then separated on a 10% SDS-PAGE gel and transferred to a nitrocellulose membrane. The membrane was blocked with 5% milk in Tris-buffered saline for 1 h and then incubated with a primary antibody against type I collagen (1∶1000, Cell Signaling Technology, Inc.) followed by IRDye secondary antibody. For internal loading control and normalization, the same membrane was striped and re-probed with anti-β-actin (1∶3000, Sigma). The bands were visualized with an ODYSSEY infrared imaging system (Li-cor Bioscience). Densitometric quantification of immunoreactive bands was performed using the software FluorChem 8800 (Alpha Innotech, San Leabdro, CA).

### Hematoxylin and Eosin (H&E) stain

Transverse sections of the urinary bladders (10 µm thickness) from all animals were stained with an H&E Stain kit according to the protocol provided by the manufacture (Richard-Allan-Scientific, Kalamazoo, MI). The sections were examined with a Zeiss brightfield microscope. Three sections were randomly chosen and four random measurements were made for each animal and averaged as one point (n). The measurement of the thickness of the bladder wall was made with software AxioImage built in the Zeiss microscope.

### Statistical analysis

Comparison between control and experimental groups was made by using one-way ANOVA followed by Dunnett's test to compare each treatment with control, or Newman-Keuls test to compare all groups. When two groups were compared, student's *t*-test was used. Differences between means at a level of p≤0.05 were considered to be significant.

## Results

### MRI examination of the urinary bladder in normal and cystitis animals

**Figure 1:**
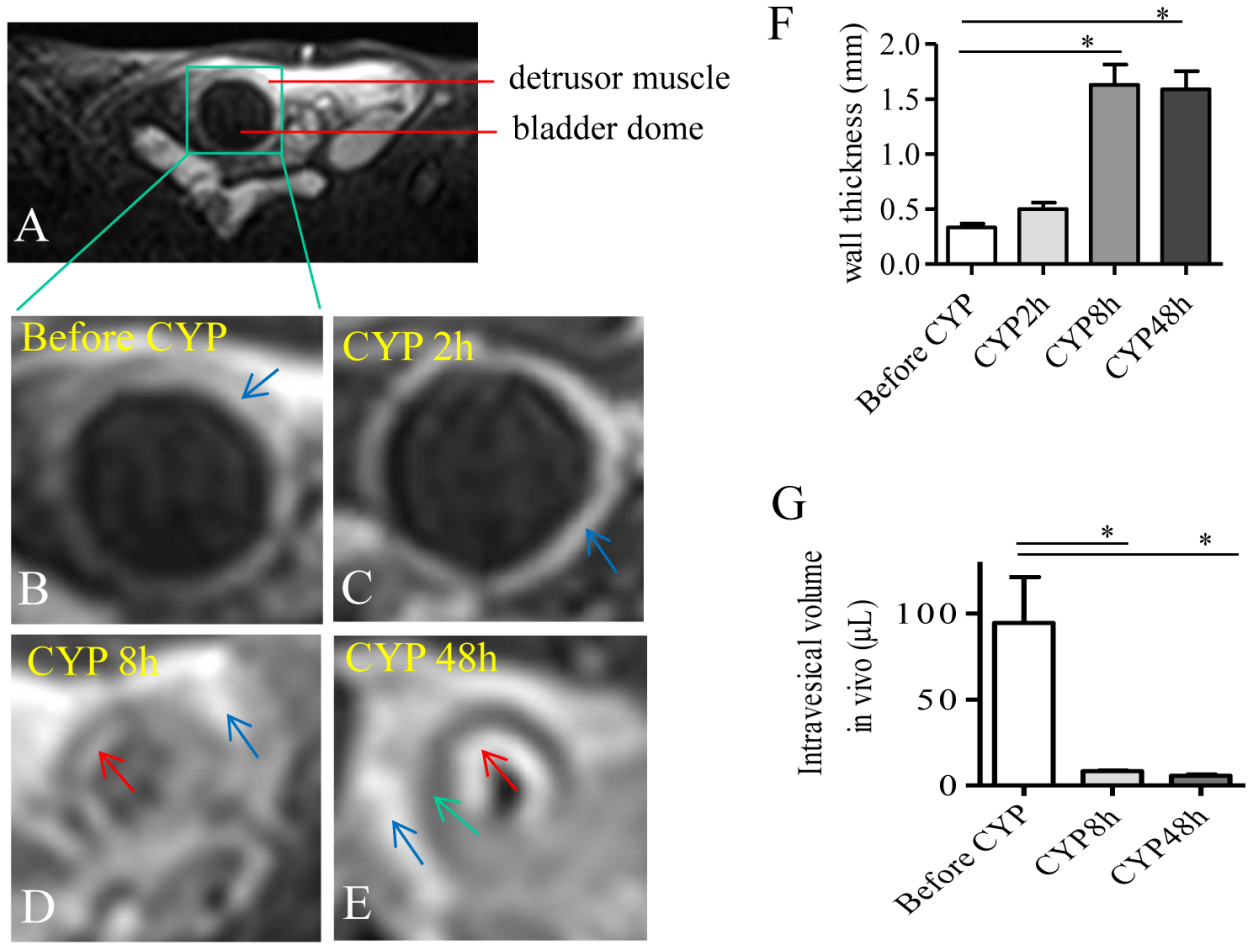
MRI visualization demonstrated an increase in the thickness of bladder wall and a decrease in the volume of bladder dome during CYP-induced cystitis. A spin-echo T2-weighted MR sequence was performed to visualize the urinary bladder in the pelvis on axial sections (A). The same animal was scanned before (B) and after CYP treatment (C-E). At 2 h after cystitis was induced, the anatomy of the urinary bladder appeared similar to control. At 8 h and 48 h post cystitis induction, the thickness of the bladder wall was significantly increased (F) which was accompanied with a decrease in intravesical volume (G). Summary results were from 5 animals before and after CYP injection. *, p<0.05.

The animals were examined under 2% isoflurane without manually expressing the urinary bladder. The axial and sagittal planes of the urinary bladder were obtained to better visualize the dome of the urinary bladder (Fig. 1A shows an axial section). A spin-echo T2-weighted MR sequence revealed that the urinary bladder in control animals demonstrated as a smooth hollow organ in the pelvis (Fig. 1B).

Our previous studies demonstrated that CYP treatment caused urinary bladder inflammation and bladder wall thickening examined ex vivo by histology and molecular approaches [6], [14]. MRI scan of CYP-treated animals provided a better visualization of the urinary bladder anatomy in vivo in live animals. At 2 h post CYP treatment the urinary bladder had similar anatomic appearance to that from control animals (Fig. 1C). At 8 h and 48 h post cystitis induction the entire wall of the urinary bladder became thicker when compared to control (Fig. 1D and Fig. 1E).

At each time point following CYP treatment, the muscular layer of the urinary bladder demonstrated low signal intensity (green arrow). There was no difference in the signal intensity emitted between the thin and thick detrusor muscular layers and the differentiation between the control and pathologic states was based on thickness. The intravesical side of the wall (red arrows) demonstrated high signal intensity in CYP-treated animals when compared to control, which indicated congestion and/or inflammation of the urinary bladder according to published criteria [39], [40], [41]. The serous membrane also demonstrated great congestion/inflammation/adhesion in CYP-treated animals when compared to control (blue arrows).

The thickness of the bladder wall including all layers of the bladder structure was measured using built-in measurement tools showing that CYP cystitis caused an increase in the thickness of the bladder wall (Fig. 1F). The intravesical volume was calculated by volume formula based on the measurement of the intravesicle radius. CYP cystitis also resulted in a decrease in bladder intravesical volume at 8 and 48 h following CYP treatment (Fig. 1G).

### MRI examination of the effects of PI3K inhibition on the urinary bladder hypertrophy

**Figure 2:**
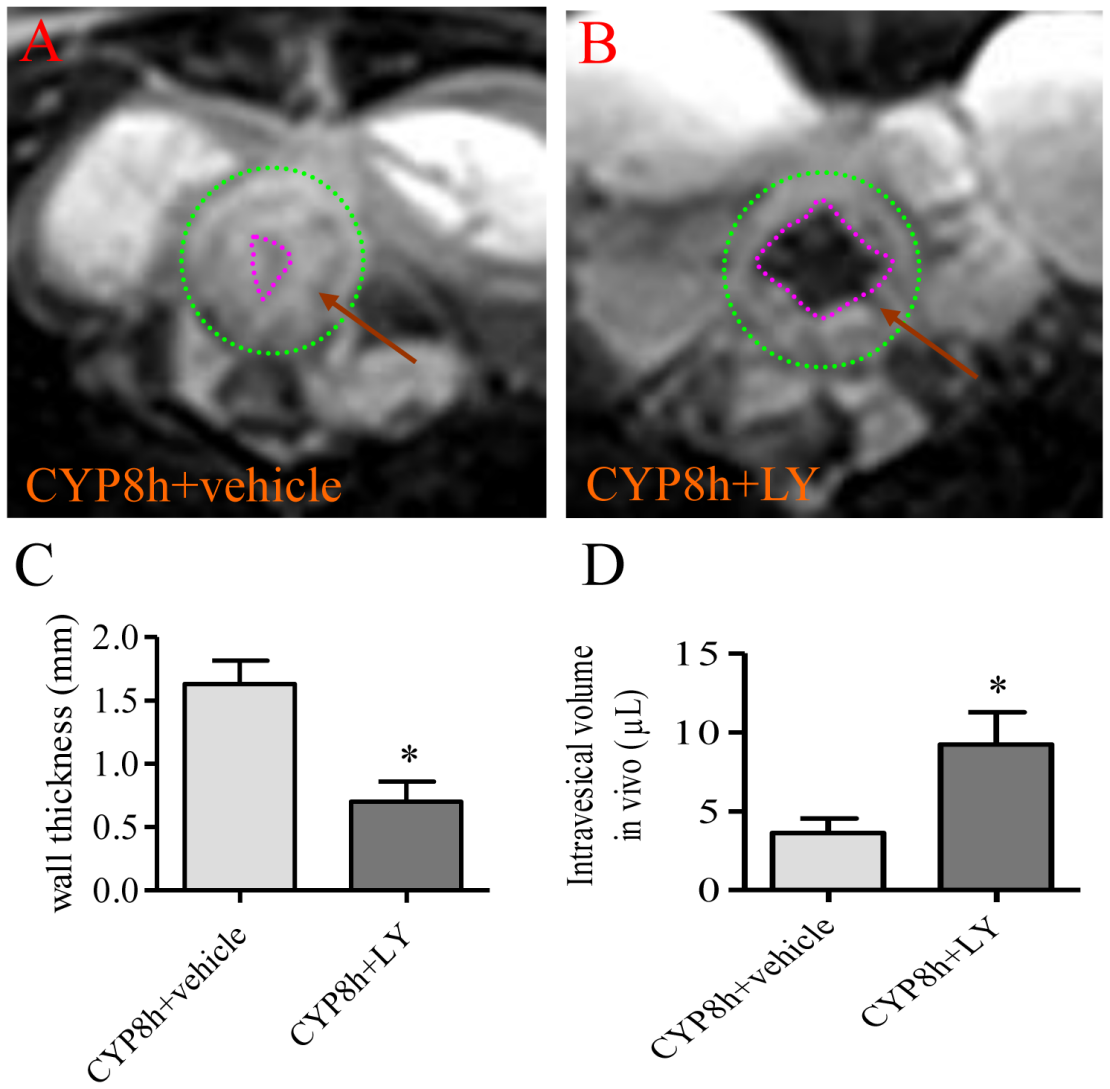
PI3K inhibition with LY294002 attenuated cystitis-induced bladder wall thickening and restored bladder capacity. The urinary bladders from cystitis animals (8 h, A) and cystitis animals treated with PI3K inhibitor LY294002 (CYP 8 h + LY, B) were visualized by MRI. Green circle indicated the outer edge of the urinary bladder (A, B); the pink circle indicated the inner edge of the urinary bladder (A, B). Treatment with LY294002 prevented bladder wall thickening caused by cystitis (C), and also increased the intravesical volume during cystitis (D). n = 4. *, p<0.05.

The PI3K/Akt pathway plays a significant role in regulating bladder function during cystitis [6], [37], [44]. In the present study, we suppressed the endogenous PI3K activity by LY294002 and examined the bladder wall thickness by MRI in order to test whether the PI3K/Akt pathway has a role in cystitis-induced bladder organ hypertrophy in vivo. Following LY294002 treatment, the thickness of the bladder wall from cystitis animals was significantly reduced (compare Fig. 2B to Fig. 2A, areas between green and red circles were bladder walls indicated by red arrows, summary data Fig. 2C). The intravesical volume was significantly enlarged in the LY294002 treated cystitis animals when compared to CYP treatment alone (compare Fig. 2B to Fig. 2A, inside the red circle marked by *, summary data Fig. 2D).

### Confirmation of the effects of PI3K inhibition on preventing cystitis-induced bladder hypertrophy by histology

**Figure 3:**
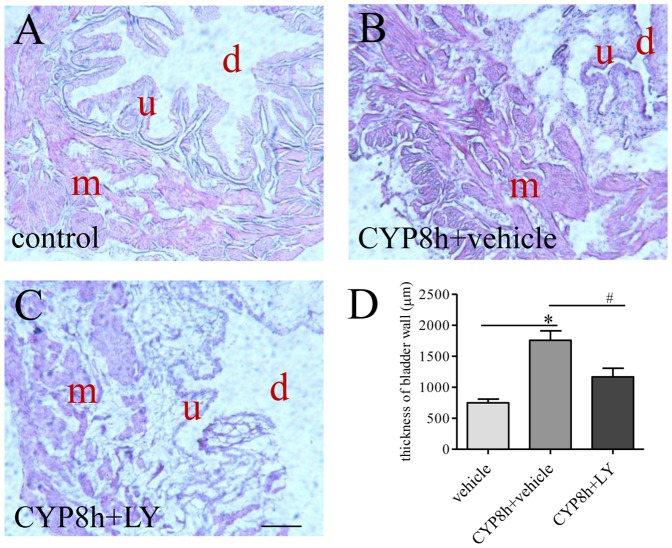
Postmortem examination by H&E showed PI3K inhibition on bladder wall thickening during cystitis. Transverse sections (10 µm) of the urinary bladder from control (A), cystitis (B) and cystitis with LY294002 (LY) treatment (C) were stained by H&E (m: muscular layer; u: urothelium; d: bladder dome). The thickness of the bladder wall was increased by cystitis (D). LY294002 treatment reduced bladder wall thickness caused by cystitis (D). Bar  = 200 µm. n = 3. *, p<0.05 vs vehicle control. #, p<0.05 vs CYP cystitis.

The effects of LY294002 on the urinary bladder hypertrophy and inflammation were further examined by histological techniques. We have previously showed that CYP cystitis increased the thickness of the bladder wall examined by H&E stain [6]. To confirm the effects of LY294002 on the thickness of the urinary bladder examined by MRI, we compared the H&E stain of the urinary bladder from cystitis animals and those animals also receiving LY294002 treatment (Fig. 3A, Fig. 3B, and Fig. 3C). Microscopic examination showed that the thickness of the muscle layer was significantly increased, and the suburothelium spaces also expanded dramatically after CYP treatment, resulting in a significant increase in the thickness of the bladder wall (compared Fig. 3B to Fig. 3A, summary data Fig. 3D). LY294002 treatment greatly reduced the thickness of the bladder wall when compared to CYP treatment alone (compare Fig. 3C to Fig. 3B, summary data Fig. 3D). It was apparent that the architecture of the urothelium was greatly damaged in the cystitis animals (compared Fig. 3B to Fig. 3A) and was recovered, in part, by LY294002 treatment (compared Fig. 3C to Fig. 3B and Fig. 3A).

### PI3K inhibition reversed cystitis-induced fibrosis and inflammatory response thereby reducing bladder hypertrophy

**Figure 4:**
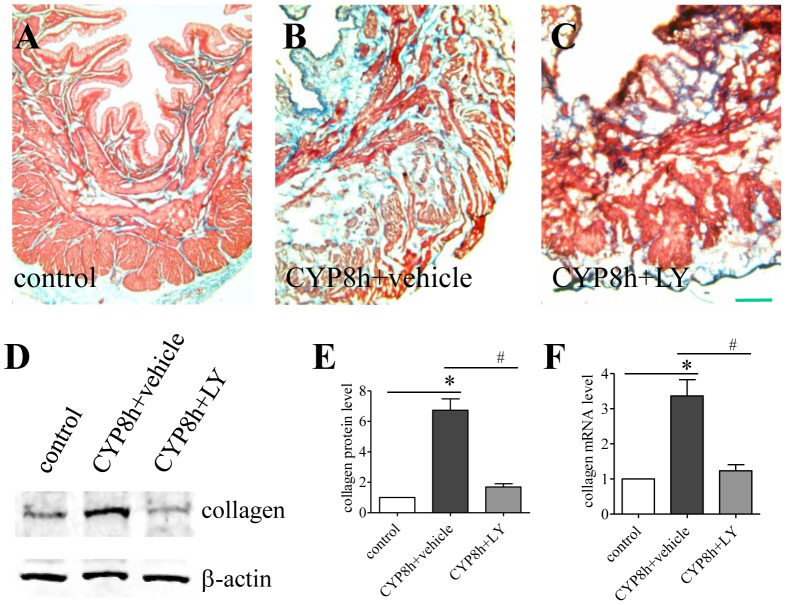
PI3K inhibition reduced collagen up-regulation by cystitis. Trichrome stain (A-C) showed an increased amount of extracellular matrix built-up in the urinary bladder at 8 h of cystitis (B, blue stain). LY294002 (LY) treatment reduced the level of extracellular matrix content in the urinary bladder of cystitis animals (C). Western blot (D) with a specific antibody recognizing type I collagen showed similar results that cystitis increased collagen protein expression which was attenuated by LY294002 treatment (E). Real-time PCR demonstrated that cystitis-induced type I collagen mRNA up-regulation was also inhibited by LY294002 treatment (F). n = 3. Bar n = 200 µm. *, p<0.05 vs vehicle control. #, p<0.05 vs CYP cystitis.

Fibrosis is one of the major factors that contribute to urinary bladder hypertrophy [1]. We also showed that type I collagen, a main component of the extracellular matrix, was up-regulated in the urinary bladder with cystitis [6]. In bladder explant culture, the PI3K/Akt pathway was involved in NGF-induced type I collagen production [6]. Thus, we further examined whether the endogenous PI3K activity had a role in regulating type I collagen production thereby regulating bladder cytology and functionality. Trichrome stain showed an increase in the levels of extracellular matrix in the urinary bladder during cystitis (blue stain, compare Fig. 4B to Fig. 4A). When cystitis animals were treated with LY294002, the level of extracellular matrix was lower than those from cystitis animals (compare Fig. 4C to Fig. 4B). Western blot analysis of type I collagen protein level confirmed these observations (Fig. 4D) showing that suppression of endogenous PI3K activity reduced cystitis-induced type I collagen up-regulation in the urinary bladder (Fig. 4E). The PI3K/Akt pathway also regulated collagen expression at the transcriptional level (Fig. 4F).

**Figure 5:**
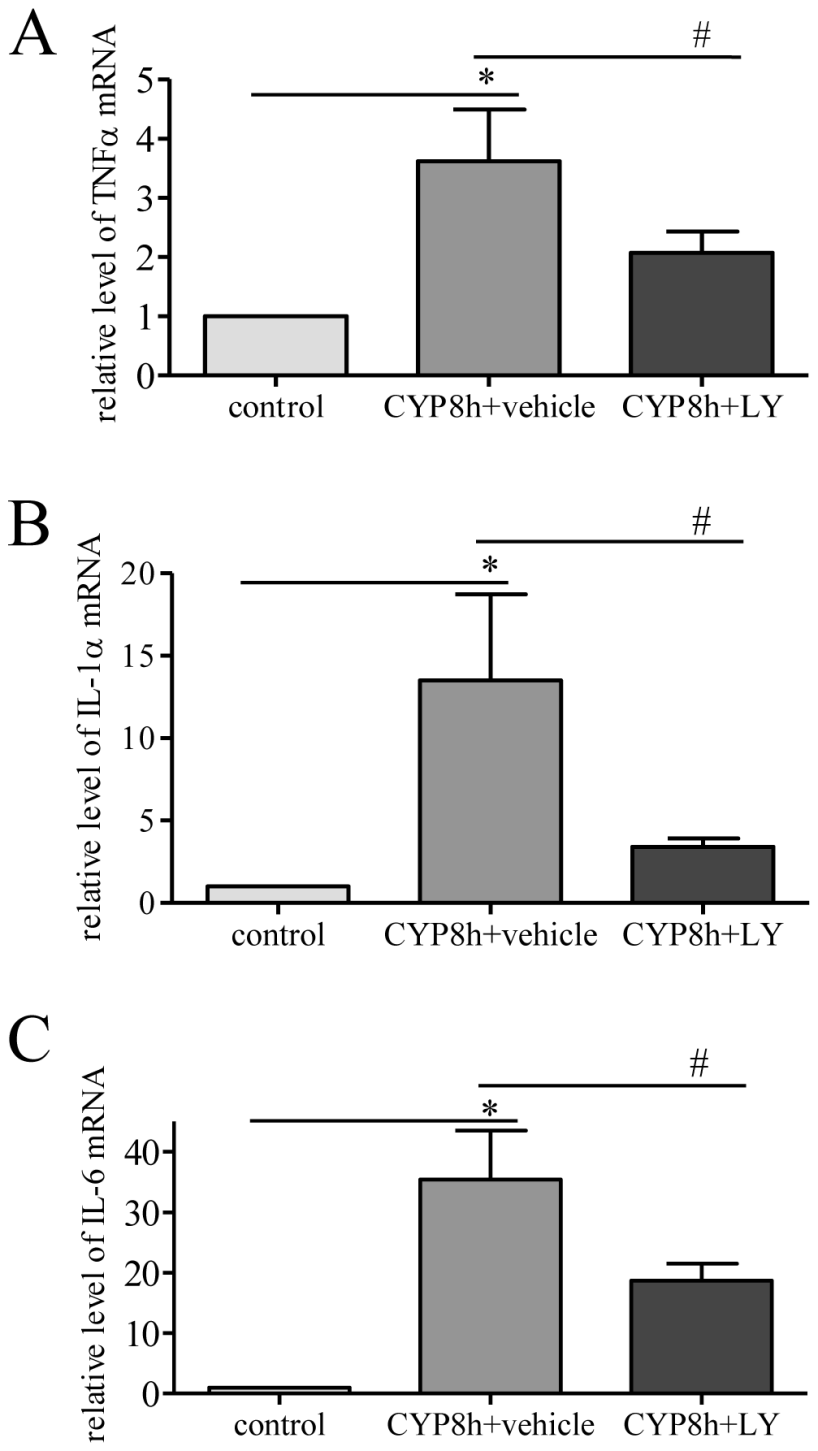
Cystitis-induced up-regulation of pro-inflammatory factors was attenuated by LY294002 treatment. The levels of tumor necrosis factor alpha (TNFα) (A), interleukin 1α (B) and IL-6 (C) were up-regulated by cystitis examined at 8 h post CYP injection. LY294002 (LY) treatment reversed inflammatory responses in the urinary bladder caused by cystitis. n = 3. *, p<0.05 vs vehicle control. #, p<0.05 vs CYP cystitis.

Inflammation of the urinary bladder was characterized by the production level of pro-inflammatory factors: tumor necrosis factor (TNF)α (Fig. 5A), interleukins IL-1α (Fig. 5B) and IL-6 (Fig. 5C). CYP cystitis significantly increased the expression level of these factors in the urinary bladder. LY294002 treatment reduced the up-regulation of the pro-inflammatory factors in the urinary bladder (Fig. 5). These results suggested that the PI3K/Akt pathway was able to regulate inflammatory responses in vivo during cystitis.

**Figure 6:**
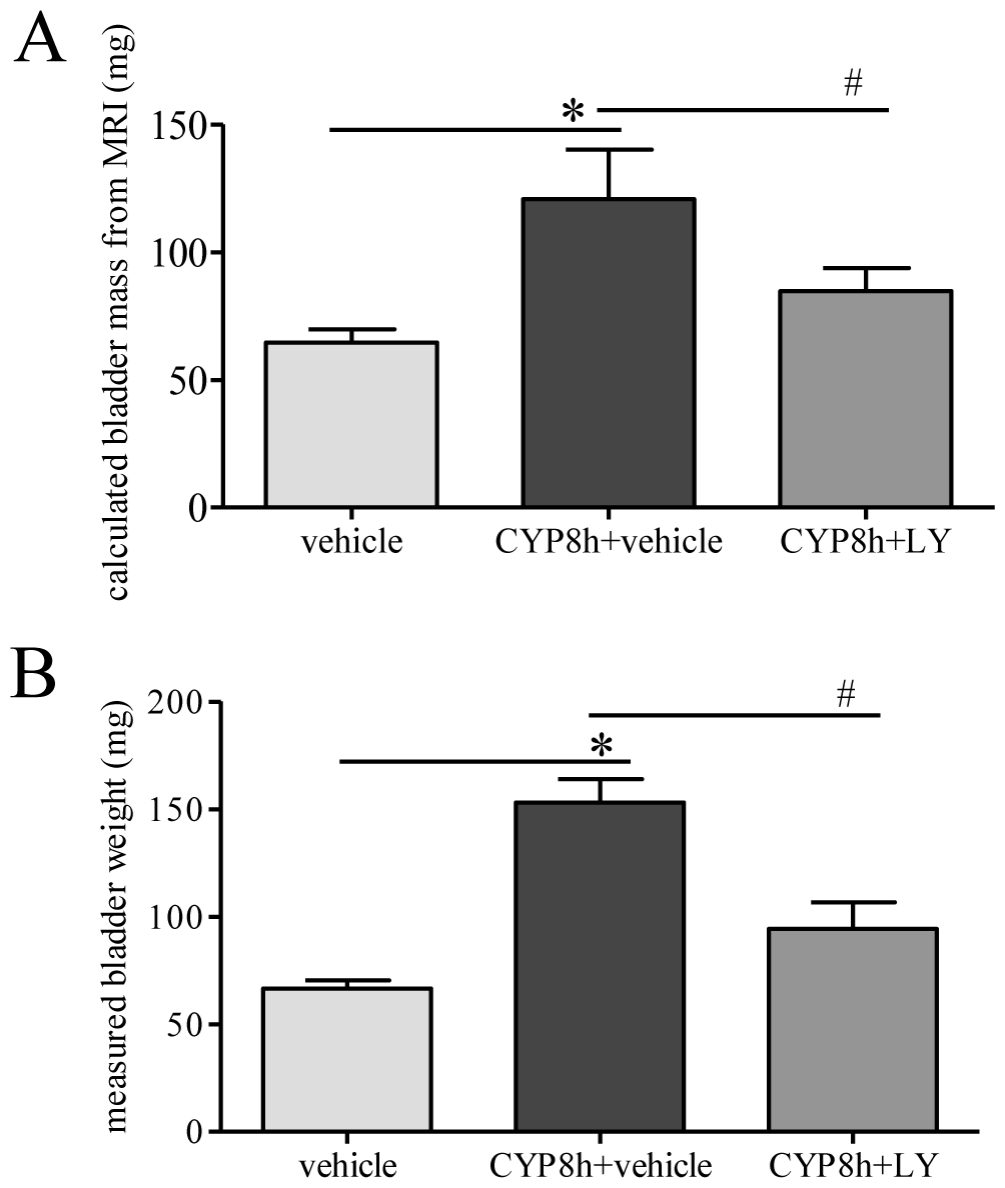
PI3K inhibition decreased cystitis-induced increment of bladder weight measured in vitro and in vivo. The bladder mass was calculated by analyzing MRI data and using a formula described in the methodology (A). Postmortem measurement of the bladder weight showed an increase in cystitis animals when compared to control (B); this increment was reduced by LY294002 (LY) treatment (B). n = 4–5. *, p<0.05 vs vehicle control. #, p<0.05 vs CYP cystitis.

When the collagen production and inflammation were suppressed by LY294002 treatment, the size of the urinary bladder was subsequently reduced in cystitis animals when compared to CYP treatment alone (Fig. 6). Using the formula described in the method section, we calculated the bladder mass in vivo based on the MRI results and demonstrated an increase in the CYP groups and a reduction in LY294002 treated groups (Fig. 6A); these numbers were very similar to the wet weight of the urinary bladder measured ex vivo (Fig. 6B) suggesting that MRI is reliable in the measurement of bladder hypertrophy in situ.

## Discussion

The greatest advantage of magnetic resonance is its ability to differentiate between a normal urinary bladder, and other pathologic conditions affecting the urinary bladder, including inflammatory, congestive and neoplastic processes without sacrificing the animals [39]. The present study utilized this in vivo live technique to monitor the urinary bladder in real-time and examined the anatomic changes in the urinary bladder during cystitis in rats and also examined the effects of the PI3K inhibition on the urinary bladder. MR images showed that cystitis increased the overall mass of the urinary bladder by increasing the thickness of the bladder wall and increasing the inflammatory responses shown as increased magnetic intensity of the bladder lining. The intravesical volume of the urinary bladder was markedly decreased following CYP treatment. However, intervention with a PI3K inhibitor LY294002 reduced the bladder mass, the thickness of the bladder wall and partially restored the capacity of the urinary bladder. Ex vivo histology confirmed the MRI observation showing the ability of the PI3K inhibitor in reversing urinary bladder thickening caused by cystitis. Further examination revealed that the effects of the PI3K inhibition on reducing urinary bladder hypertrophy were due to its ability in decreasing the degree of fibrosis and inflammatory responses in the urinary bladder.

MRI techniques have been widely used in humans to examine the internal structure of soft tissues in normal and diseased states [45], [46]. The magnetic field acts upon protons found in water within the soft tissue which sends out signals as radio waves, thus it is especially helpful to collect pictures of internal organs and muscles that do not show up on x-ray examination. This technique can be used to scan tissues in the body including pelvic organs [47]. As for the urinary bladder which contains a large volume of water in the dome and produces high background signals during visualization of the wall, T2-weigthted MR imaging is often used for its low signal intensity to differentiate the tissues from the urine and the perivesical fat [48], [49]. In the present study, we also used T2-weighted MRI to examine the thickness of the bladder wall. To better distinguish the tissue anatomy, we applied water-suppression technique to enhance the signal of the soft tissue by extensively lowing the signal in the dome. This gave us a better visualization of the urinary bladder, and provided means to identify the thickness of the bladder wall. MRI techniques have been successfully used to detect bladder wall thickness in humans and experimental animals in normal and diseased states [39], [40], [49], [50]. MRI results show an increase in the thickness of the bladder wall in patients with BOO or cystitis [40]. In this study in a rat model of chemically-induced cystitis, we found that the MRI results were consistent to those examined ex vivo by histology, suggesting that MRI can be used for this disease model to detect the real-time changes of the urinary bladder and study drug efficacy during treatment. In some of our studies, we attempted to manually empty the urinary bladder and found that the bladder wall was completely collapsed which made it very difficult to examine the thickness (data not shown). This is consistent to published studies demonstrating that evaluation of bladder wall thickening with MRI must require some degree of urinary bladder distention [39], [40]. During MRI, the urinary bladder is not fully distended and is not ready for micturition. Thus the intravesical volume obtained by MRI is much smaller than those obtained by measuring the amount of urine voided through micturition [51].

Cystitis-induced bladder hypertrophy is accompanied by collagen up-regulation [6] and increased inflammatory responses in the urinary bladder. Anatomic and molecular studies in the present paper demonstrate that the PI3K pathway is involved in cystitis-induced bladder hypertrophy by up-regulating collagen mRNA and protein levels, and by increasing the production of pro-inflammatory factors in the urinary bladder. Activation of PI3K can lead to the activation of Akt, a downstream effector that is activated in the urinary bladder during cystitis [6]. It is reported that the PI3K pathway is involved in collagen production in culture [6]. The regulation of collagen by the PI3K pathway is also found in cultured human dermal fibroblasts [52], human retinal pigment epithelial cells [53], and human tenon's fibroblasts [54]. Our study further demonstrates that the activation of the PI3K pathway also leads to collagen up-regulation in vivo because inhibition of the endogenous PI3K activity reverses cystitis-induced type I collagen up-regulation in the urinary bladder, ultimately reverses cystitis-induced bladder wall thickening.

The elevated inflammatory response is another major contributing factor to urinary bladder hypertrophy. Cystoscopy findings show the presence of glomerulations and Hunner's ulcers on the bladder wall, scarred or stiff wall with low compliances and reduced bladder capacity in cystitis patients [55]. Cytokines, chemokines, prostaglandins, adenosine systems and growth factors are also examined in the urinary bladder with OAB, DO or cystitis [1], [2], [20], [56]. In response to bladder irritation, the innermost urothelium layer undergoes considerable injury with increased apoptosis and accumulation of inflammatory cells such as mast cells, neutrophils and macrophages accompanied with release of the inflammatory mediators [56]. The subsequent actions of these factors are to modify detrusor smooth muscle mitosis and contractility thus altering the activity of gene expression and ion channels, regulating cell cycle events and increasing protein production and deposition of extracellular matrix leading to bladder hypertrophy. In CYP-induced bladder inflammation and hypertrophy shown in the present study, the levels of pro-inflammatory factors IL-1α, IL-6 and TNFα are increased; which is attenuated by administration of the PI3K inhibitor LY294002. These results suggest that the PI3K pathway also has a role in regulating inflammatory responses in the urinary bladder.

In summary, the present studies utilizing imaging techniques and molecular and histologic tools demonstrate that the PI3K has a crucial role in regulating urinary bladder hypertrophy examined in a cystitis rat model induced by CYP. The molecular mechanisms underlying the PI3K regulation of bladder hypertrophy lies in its ability in the regulation of type I collagen up-regulation in vivo and its role in regulating the production of pro-inflammatory factors. Intervention of the PI3K pathway may be effective in treatment of bladder hypertrophy and associated diseases. In the examination of bladder wall thickening in normal and diseased states, MRI can be used for in vivo visualization and can also be used as a pre-clinical tool in the evaluation of drug effects.
